# Introducing eHealth strategies to enhance maternal and perinatal health care in rural Tanzania

**DOI:** 10.1186/s40748-017-0042-4

**Published:** 2017-01-19

**Authors:** Angelo Nyamtema, Nguke Mwakatundu, Sunday Dominico, Mkambu Kasanga, Fadhili Jamadini, Kelvin Maokola, Donald Mawala, Zabron Abel, Richard Rumanyika, Calist Nzabuhakwa, Jos van Roosmalen

**Affiliations:** 1 Thamini Uhai Program, Dar es Salaam, Tanzania; 2 Tanzanian Training Centre for International Health, Ifakara, Tanzania; 3 Saint Francis University College for Health and Allied Sciences, Ifakara, Tanzania; 4 Catholic University of Health and Allied Sciences, Mwanza, Tanzania; 5 Maweni Regional Hospital, Kigoma, Tanzania; 6 Leiden University Medical Centre, Leiden, The Netherlands; 7 Athena Institute, VU University Amsterdam, Amsterdam, The Netherlands

**Keywords:** EHealth, eLearning, Teleconference, Teleconsultation, Rural settings, Maternal health, Tanzania

## Abstract

**Background:**

Globally, eHealth has attracted considerable attention as a means of supporting maternal and perinatal health care. This article describes best practices, gains and challenges of implementing eHealth for maternal and perinatal health care in extremely remote and rural Tanzania.

**Methods:**

Teleconsultation for obstetric emergency care, audio teleconferences and online eLearning systems were installed in ten upgraded rural health centres, four rural district hospitals and one regional hospital in Tanzania. Uptake of teleconsultation and teleconference platforms were evaluated retrospectively. A cross sectional descriptive study design was applied to assess performance and adoption of eLearning.

**Results:**

In 2015 a total of 38 teleconsultations were attended by consultant obstetricians and 33 teleconferences were conducted and attended by 40 health care providers from 14 facilities. A total of 240 clinical cases mainly caesarean sections (CS), maternal and perinatal morbidities and mortalities were discussed and recommendations for improvement were provided. Four modules were hosted and 43 care providers were registered on the eLearning system. For a period of 18–21 months total views on the site, weekly conference forum, chatroom and learning resources ranged between 106 and 1,438. Completion of learning modules, acknowledgment of having acquired and utilized new knowledge and skills in clinical practice were reported in 43–89% of 20 interviewed health care providers. Competencies in using the eLearning system were demonstrated in 62% of the targeted users.

**Conclusions:**

E-Health presents an opportunity for improving maternal health care in underserved remote areas in low-resource settings by broadening knowledge and skills, and by connecting frontline care providers with consultants for emergency teleconsultations.

## Background

Tanzania has one of the highest maternal mortality ratios (MMR 398/10^5^ live births) and one of lowest densities of skilled health care providers in the world, an element which is critical to enhance maternal and perinatal health [1]. Skilled health care workers (i.e. physicians, associate clinicians, nurse-midwives, and laboratory, radiology and pharmaceutical technicians) fill only 35% of the requirement, and only 55% of births in rural areas are assisted by skilled personnel compared to 87% in urban areas. This suggests not only a severe crisis of human resources for health, but also serious health inequities across the country [2, 3]. Like many other countries, Tanzania has embarked on task-sharing by using less trained mid-level care providers including clinical officers (associate clinicians) and assistant medical officers (advanced associate clinicians) [4, 5]. The World Health Organization (WHO) defines associate clinician as a professional with basic competencies to diagnose and manage common medical, maternal, child health and surgical conditions [6]. Advanced level associate clinician is defined as a professional with advanced competencies to diagnose and manage the most common medical, maternal, child health and surgical conditions, including obstetric and gynaecological surgery (e.g. caesarean sections). Although evidence shows that mid-level care providers can perform related tasks, task sharing must be aligned with broader strengthening of knowledge and clinical skills, mentorship and support if sustainable provision of quality health care is to be achieved [7].

Worldwide consensus exists that eHealth, the use of information and communication technologies (ICT) for health, has the potential to significantly improve management of patients, conducting research, educating health care providers, tracking diseases and monitoring public health [8]. EHealth can be used to improve knowledge, competencies, accountability and effectiveness; mentorship provision and health system’s ability to manage its commodities, equipment and health care workers [9]. Increasing uptake of mobile ICT in Tanzania constitutes new opportunities to support maternal and perinatal health care interventions. Tanzania’s eHealth strategy 2013, through its well-articulated strategic objectives, emphasizes the need to improve quality of health service delivery in rural settings through eHealth solutions [3].

Recognizing the potential of ICT in addressing challenges in provision of quality maternal health care, the Thamini Uhai program designed and implemented an eHealth platform with a goal to support national efforts to address the crisis of skilled care providers and improve maternal health care delivery [3]. Our platform aimed at improving knowledge and clinical decision-making skills of mid-level care providers, support emergency care and formulate an eHealth model solution for maternal health care in underserved rural settings. This study describes the Thamini Uhai program’s best practices, gains and challenges of implementing eHealth for maternal health care in extremely remote and rural Tanzania.

## Methods

### Project areas

The project established an eHealth platform to support fifteen health facilities (ten upgraded health centres [HC], four rural district hospitals and one regional referral hospital) located in three regions (Kigoma, Morogoro and Pwani) in Tanzania. Nine upgraded HC were located in the hardest to reach rural areas in seven districts. Upgrading of HC involved constructing and equipping maternity blocks, operating theatre, laboratories, staff houses and installing solar panels, standby generators and water supply systems, and training associate clinicians in CEmONC and anaesthesia. The details of the support other than the eHealth platform have been reported elsewhere [10].

### ICT infrastructure design

The eHealth platform had three components: 1) mobile teleconsultations for obstetric emergency care; 2) an audio teleconferencing model; and 3) an online eLearning platform. The mobile teleconsultation platform was designed to enable health care providers, working in Thamini Uhai-supported facilities, to call experienced obstetricians and discuss obstetric emergencies in which further guidance or advice was needed to help providers make the right decisions. Health care providers in the supported facilities and consultant obstetrician/gynaecologists were connected through toll-free mobile phones by way of a closed user group. Duty rosters for consultant obstetricians were developed and all calls made for consultation were documented. This service was available 24 hours a day, seven days a week.

An audio teleconferencing model was established in 2012 and health care providers in the supported facilities, consultant obstetrician/gynaecologists, and anaesthesiologists were routinely connected through toll-free mobile phones by way of a closed user group. This model applied the concept of case study-based learning, a form of problem-based learning, using challenging obstetric cases encountered over the previous week to increase knowledge and understanding and develop generic skills and encourage self-reflection in maternal health care. During the teleconference one person from each facility reported the weekly number of deliveries, caesarean sections, vacuum-assisted vaginal deliveries, number and reasons for obstetric referrals. Severe morbidities, fresh stillbirths, very early neonatal and maternal deaths were reported in length and management and justifications (indications) for interventions were discussed. Selection of these cases was based on the fact that they might be preventable based on changes in care, resources, education, or medical access. The contents of these teleconferences were documented and disseminated to the relevant stakeholders for action-oriented feedback.

An online eLearning platform was established in 2014. The learning sessions were hosted in a web-based eLearning application called Moodle. To address the challenge in internet connectivity in rural settings, Multi-Protocol Label Switching (MPLS) Virtual Private Network was installed in all supported facilities to enable users in remote facilities to connect with the application server in Dar es Salaam through laptops. Network Address Translation (NAT) enabled accessibility of the application through the web. Each facility was equipped with at least one laptop through the local contact person.

A total of four sessions were hosted in the eLearning platform. These included caesarean section, spinal anaesthesia (which included two tailor-made education films), management of postpartum haemorrhage and neonatal resuscitation. This ‘virtual classroom’ also enabled health care providers to access presentations and up-to-date peer-review journal articles. The system also allowed registered users to use integrated online fora to share clinical challenges and successes with fellow colleagues and senior experts across the 15 health facilities.

### Study design, study population and sampling technique

A retrospective study design was applied to evaluate the mobile teleconsultation platform and audio teleconferencing model. A cross sectional descriptive study design was applied to evaluate the performance and adoption of online eLearning platform. The study population for online eLearning platform were health care providers working in supported health facilities. Only those health care providers who were available on the day of study were included in the study. Considering the diversity of the topics of learning sessions in the online eLearning platform, purposive selection of health care providers for interview was preferred and applied. Because of the time limitations placed on the study, at least one care provider was selected from each health facility to demonstrate skills on using eLearning platform.

### E-learning system evaluation framework

**Fig. 1:**
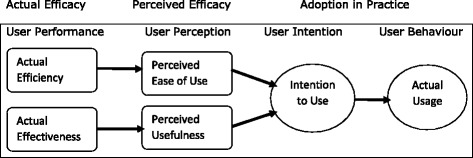
A theoretical model for evaluating information systems design methods

To evaluate performance and adoption of the eLearning system the Method Evaluation Model (MEM) was used. MEM was chosen because it incorporates all aspects of evaluation including user performance, perceptions of usefulness, intentions to use and user behaviour towards the system (Fig. 1) [11].

In the MEM model, efficiency is measured by the extent to which use of the eLearning system by health care providers is effort free, whereas actual effectiveness is measured by the extent to which use of the eLearning system improves the quality of learning. Perceived ease of use and perceived usefulness represented user’s perceptions about the usefulness and ease of use of the eLearning platform. The third construct was the intention to use, which is the extent to which a health care provider intends to use the eLearning system in the future for learning purposes. The last construct measured actual usage of the system.

### Data collection instruments

Four types of instruments for data collection were used in this study.

#### Instrument 1: Data collection instrument for the mobile teleconsultation platform

This instrument was used to extract the number of calls made, obstetric indications (types of emergencies) for calling, type of support provided and outcome.

#### Instrument 2: Data collection instrument for audio teleconferencing model

This instrument was used to collect data on the number of health facilities and health care providers who participated in each teleconference, frequency of connectivity interruptions, categories of cases (morbidity, mortality, and procedures) presented and discussed.

#### Instruments 3.1 and 3.2: Data collection tools for e-Learning

These were developed to gain as much information as possible regarding design and impact of the eLearning system on improving learning and health outcomes. Instrument 3.1: end-user questionnaire, which aimed at assessing provider’s awareness, knowledge, perception and practice of using the e-Learning platform. Instrument 3.2: Review and analysis of the general and session specific activity report worksheets. These data were obtained by commanding the reports on the eLearning platform.

### Data collection

Data collection tools for mobile teleconsultation and audio teleconferencing platforms were used to extract information from the available records. Data collection for eLearning platform involved a team of experts who visited the health facilities to evaluate user performance, perceptions and intentions, and user behaviour towards the installed system.

#### Effectiveness of the eLearning system

To investigate users’ effectiveness of using the eLearning platform, health care providers were required to demonstrate the use of the eLearning system when the investigator observed. Then the investigator, guided by instrument 3.1, observed how effectively health care providers used the module and whether they had completed, and/or had learnt new things. Furthermore, through interviews, the investigation aimed at determining whether health care providers used in practice the knowledge they had learned through the eLearning system.

#### Efficiency of the eLearning system

To investigate efficiency on using the eLearning platform health care providers were required to demonstrate use of the system in the presence of an observer. Seven tasks were used to assess efficiency: enter user name and password, identify features within the platform, ability to use features within the platform (site news, chatroom, learning resources), identify the four sessions in the platform, open one of the sessions, navigate through the sessions, and perform quiz questions within the platform.

#### Perception-based variables

Perceived ease of use is the degree to which a person believes that using the eLearning system is free of effort. In order to investigate users’ perception of the eLearning platform, we asked the participants to grade the system based on how easy it was to use the system. We scaled responses on a Likert scale from 1 to 5, i.e. very difficult, difficult, neutral, easy and very easy.

#### Intention to use variables

Participants were asked questions designed to assess their intentions to use the eLearning system in the future for learning purposes.

#### Actual usage variables

To investigate actual usage of the system we retrieved data from instrument 3.2, the platform general activity and session specific reports obtained by commanding the reports on the e-Learning platform. The data included user access to the website, user participation in chats, posting and viewing various learning contents.

### Data analysis

Data were cleaned and consistency checks were done using Microsoft Excel. Actual effectiveness, perceived efficacy and adoption in practice of the eLearning system were analysed using Microsoft Excel and summarized in proportions.

## Results

### Teleconferencing and mobile teleconsultation platforms

A total of 33 teleconferences were conducted in 2015 and 40 health care providers from 14 supported facilities took part. Participants included 14 (35%) assistant medical officers, 13 (32.5%) nurse-midwives/clinical officers trained in anaesthesia, and 13 (32.5%) additional nurse-midwives. Health facility participation ranged from 2 to 6 per teleconference with an average of 4 facilities per teleconference. The facility recording the highest level of participation was Kibiti HC in Pwani region, which participated in 82% (27) of the teleconferences (Table 1). Nyenge HC in Kigoma region did not participate in any of the teleconferences because of poor connectivity.

**Table 1:** Frequency of participation of health facilities and connectivity interruptions during the teleconferences

Health Facility	Frequency of facility participation *n* (%)	Number of care providers participated at least once	Frequency of connectivity interruptions during teleconference *n* (%)
Health Centres			
Kibiti	27 (82%)	7	0 (0%)
Ujiji	15 (45%)	3	0 (0%)
Nguruka	12 (36%)	5	1 (8%)
Mtimbira	14 (42%)	2	2 (14%)
Mabamba	12 (36%)	4	1 (8%)
Kakonko	8 (24%)	1	0 (0%)
Mlimba	11 (33%)	6	2 (18%)
Buhingu	5 (15%)	2	1 (20%)
Mwaya	6 (18%)	1	0 (0%)
Nyenge	0 (0%)	0	0 (0%)
District Hospitals			
Kibondo	2 (6%)	2	0 (0%)
Utete	12 (36%)	4	0 (0%)
Mahenge	11 (33%)	1	1 (9%)
Kasulu	1 (3%)	1	0 (0%)
Kigoma (Maweni) RRH	2 (6%)	2	0 (0%)

Note: *RRH* regional referral hospital

A total of 240 clinical cases were presented and discussed during the teleconferences. These included 7 (3%) maternal deaths, 24 (10%) perinatal deaths (fresh stillbirths and early neonatal deaths), 54 (23%) maternal and newborn morbidities and 138 (57%) caesarean sections, 12 (5%) vacuum-assisted vaginal deliveries and 5 (2%) cases of ectopic pregnancy, breech deliveries and internal podalic version for a retained second twin. Commendable performances and factors related to substandard care within the facilities were identified, discussed and recommendations for improvement were provided. During teleconferences a remarkable proportion of severe morbidities and maternal and/or perinatal deaths were attributed to inadequate, inappropriate interventions and delayed decisions. These cases were audited during physical supervision visits to the facilities and the results have been reported elsewhere [10, 12].

A total of 38 emergency teleconsultations (calls) were received and attended by consultant obstetricians/gynaecologists in 2015. These included complications of pregnancy and labour, medical conditions in pregnancy and abnormalities of menstruation. Advice on how to manage the patients was provided. A total of 33 (87%) patients were successfully managed locally and the rest (13%) were stabilized and referred to higher facilities.

### Online eLearning system: views and performance

A total of 43 health care providers were registered as users and were oriented on the eLearning platform at the time of its establishment. For a period of 18 to 21 months the total views on the site ranged between 106 and 1,438 among the different options of weekly conference forum, project chatroom, caesarean section resources, spinal anaesthesia resources and videos for various topics (Table 2).

**Table 2:** Summary of Thamini Uhai’s eLearning system utilization from its establishment in September 2014 to May 2016.

Activity	Date of posting into system	Views as of 31 May 2016
Site views	2 Sept 2014	1,438
Weekly conference forum	4 Oct 2014	487
Project chatroom	4 Oct 2014	329
Caesarean section resources	17 Nov 2014	184
Spinal anaesthesia resources	17 Nov 2014	121
Video		
Introduction on caesarean section	17 Nov 2014	208
Caesarean section procedure	17 Nov 2014	150
Introduction on spinal anaesthesia	17 Nov 2014	106
Spinal anaesthesia procedure	17 Nov 2014	125
Modules^a^		
Postpartum haemorrhage	3 Jul 2015	38
Caesarean section	20 Oct 2014	36
Spinal anaesthesia for caesarean section	20 Oct 2014	36
Birth asphyxia and neonatal resuscitation	4 Jul 2015	36

^a^The number of views and viewers were those made by registered care providers from the supported facilities

#### Actual effectiveness of the eLearning system

Almost six months after orientation, 20 care providers (47% of the registered users) from the supported health facilities were interviewed and their competencies in using the system were assessed. Among these 10 (50%) were assistant medical officers (advanced associate clinicians), one was a clinical officer (associate clinician), seven were nurse-midwives, and two were anaesthetists.

**Fig. 2:**
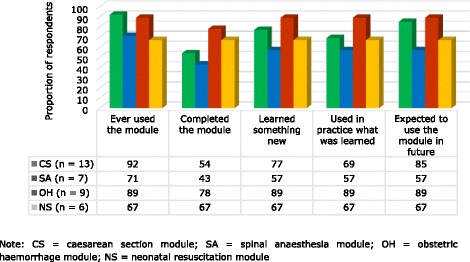
The effectiveness of the eLearning system on caesarean section, spinal anaesthesia, obstetric haemorrhage and neonatal resuscitation modules

Health care providers were grouped based on the scope of tasks they routinely performed with regards to emergency obstetric care, caesarean section and anaesthesia, and then were interviewed on the hosted modules. Between 43% and 78% of the respondents reported to have completed the modules, while 57% - 89% acknowledged to have learnt something new after reading the modules, utilized in clinical practice the lessons learned and expected to use the modules in future (Fig. 2).

Specifically, the skills that the care providers reported to have acquired through the eLearning included active management of third stage of labour, better ways of performing caesarean section, use of condom tamponade for controlling postpartum bleeding, proper preparation of pregnant women before CS and proper positioning of a pregnant woman during and after provision of anaesthesia.

#### Actual efficiency of the eLearning system

**Fig. 3:**
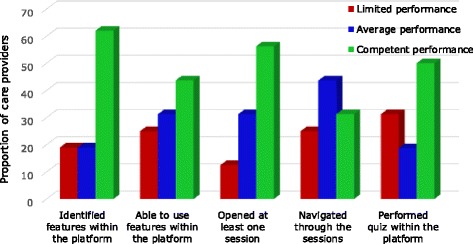
Level of efficiency of health care providers in using the eLearning system

Sixteen health care providers were involved in the assessment of competencies in using the system. Each care provider was individually assessed whether she/he was able to: identify features within the platform; open at least one session; navigate through the sessions; and perform a quiz within the platform. Competencies in performing these task were demonstrated by 5 – 10 (i.e. 31–62%) care providers (Fig. 3). As expected, competencies were demonstrated more in the simplest use-features of the system, like identification of features within the platform (62%), rather than in complex features like navigating through the sessions (31%).

### Perceived efficacy and adoption in practice of the eLearning system

Out of twenty respondents who were interviewed for this construct, 13 (65%) perceived the eLearning system to be easy to use, whereas 3 (15%) found the system to be difficult to use and 4 (20%) were neutral. None of the respondents perceived the system to be very difficult or very easy to use. The eLearning platform was positively perceived by health care providers as one of the best platforms for sharing clinical experiences; suitable for Continuing Professional Development; inspiring the user to keep on learning; providing a good opportunity for self-assessment, learning knowledge and skills that were neither taught in medical colleges nor learnt in work settings; and that the system reminded the providers of knowledge gained in medical schools. Findings indicated that 12 (60%) of the respondents had the intention to use the system in the future whereas 6 (30%) were neutral.

#### Factors affecting utilization of the ICT learning platforms

Utilization of online eLearning system was affected by infrastructural and technical factors. First, there was inadequate internet and mobile phone connectivity. Second, some eLearning users had inadequate computer literacy and there was a serious lack of local IT technical support. With the exception of only one health facility there were no IT personnel to support users and the system at the local level.

## Discussion

In order to ensure quality maternal and perinatal health care delivery, knowledge and skills of health care providers play a vital role. This underscores the importance of continuing professional development and emergency care support of frontline health care providers. Understanding the limitation of knowledge and skills of mid-level care providers working independently in underserved rural settings many advocate for the application of eHealth technologies for human resources capacity building [13, 14].

### Teleconferencing and mobile teleconsultation platforms

The fact that 60% of the health centres participated in more than one third of the teleconferences, and the fact that there were few connectivity interruptions highlights the potential of the innovative audio teleconferencing platform for improving knowledge and decision-making skills, and hence the quality of maternal and perinatal health care. The teleconference platform used real event cases (real patients with problems) that were encountered over the past week for learning. Real case studies were the preferred teaching method because they enhance active engagement of learners in professional reasoning in the midst of case decision-making and allow learners to know how expert clinicians think through the case. There were no reports of any negative consequences associated with presenting real event case studies with bad outcomes suggesting acceptability of this learning method. Studies indicate that case studies are powerful and effective training tools, and are a popular mechanism to improve the adult learning experience particularly in medicine [15]. Although 38 emergency calls (teleconsultations) were made and attended by consultant obstetricians in 2015, there is still a need to strengthen and support clinical decision-making for mid-level care providers considering the short period of their training programs, limited knowledge and skills but also the gaps identified during the teleconferences. Considering that life experiences strongly affect learning, teleconsultation encouraged immediate use of newly acquired knowledge.

Integration of mobile phone technology into the health care system provided care providers with new ways of learning and opportunities to apply knowledge, thereby enhancing clinical problem-solving and enhanced accountability/personal responsibility in care. Like other studies our findings suggest that mobile phone technology can be used for continuing professional development and may enhance clinical decision making even in underserved rural areas in low-resource settings [14, 16].

### E-learning platform

#### Efficacy of the eLearning system

In this study users reported a remarkable level of effectiveness and demonstrated an appreciable degree of competencies on the e-Learning system. Specifically, e-Learning platform users reported to have acquired a wide range of clinical skills which are critical in the management of women with obstetric complications and when providing anaesthesia. Knowledge and skills acquired by the users can partly explain improved maternal and perinatal outcomes in the project areas which have been reported elsewhere [10, 12]. Our findings suggest that an e-Learning strategy can be used to refresh health care providers with current practices and support development of clinical competencies which are essential for life saving maternal and perinatal health care [17]. However, the fact that the 13–31% of care providers demonstrated limited performance in using the eLearning system suggests further investigation to determine why the system worked with some and not others. Some studies have reported that eLearning can be as effective as or even more effective than face-to-face instruction and can be more efficient if effective techniques are used, especially for the development of knowledge and critical thinking, and decision making skills [9, 18, 19].

#### Perceived efficacy and adoption in practice of the eLearning system

The fact that health care providers perceived the eLearning platform positively with regards to its usefulness and ease of use, and that they were ready to use it in the future suggest that they were satisfied and that the e-Learning platform was acceptable. Acceptability of the platform was also manifested by a significant number of views made on the weekly conference forum, caesarean section and spinal anaesthesia resource folders. Like other studies, our findings suggest that a well-designed e-Learning platform can promote and motivate learners to become more engaged with the content [17].

These results come from rural areas, suggesting that e-Learning can help to broaden the skills of existing professionals, reach those who live in geographically isolated areas and reduce costs of learning-related travel. Considering the acute shortage of care providers, eLearning is beneficial to the health care system in that health care workers do not need to leave their work places for training. E-Learning platform allows learning at work and at home, so that learners can broaden their skills whilst continuing to provide crucial services to their communities [20]. The fact that mid-level care providers working in rural areas are the backbone of almost all national health goals in most low-resource countries, it is essential to equip them with adequate clinical knowledge and skills for optimum service delivery. Like other reports, these results suggest that a wider use of eLearning might help to address the need for capacity building of frontline health care workers [14, 21, 22].

Establishing an eHealth platform in extremely remote and rural health facilities for continuing professional development and supporting obstetric emergency care is an appropriate and effective intervention, has proven feasible and is currently operational. This report is an inspiring testimony on how to integrate eHealth into the existing health care system for human resource capacity building in the most isolated remote and rural areas in low-resource countries.

#### eHealth implementation challenges in rural settings

Although these results suggest a great potential for scaling up eHealth solutions in rural Tanzania, the use of online eLearning is facing infrastructural and technical factors that need to be overcome. These factors included inadequate computer literacy, lack of local IT technical support and inadequate internet connectivity. To optimize eLearning systems for continuing professional development, basic computer application training programs should be introduced and where available strengthened in educational curricula in secondary schools and colleges. The fact that availability of broadband in rural sub-Saharan Africa is still restricted, learning content needs to be available both online and offline. Local health facilities in remote settings can be outfitted with desktop computers and laptops where eLearning modules for essential skills updates can be uploaded.

### Limitations of the study

This study describes considerable practices on eHealth for supporting maternal and perinatal health care but does not robustly evaluate the impact of these interventions on health outcomes. The impact of these interventions on health outcomes was not evaluated because the project combined a wide range of approaches to improve knowledge, skills including face-to-face training which were also conducted during on site supportive supervision [10]. This study also does not adequately evaluate effectiveness and efficiency of the eHealth strategies, given the methodological designs (convenience and purposive sampling, and the lack of control groups).

## Conclusions

This article highlights considerable practices and gains made in using eHealth strategies for maternal and perinatal health care and offers them as a model that Ministries of Health in low-resource countries and international agencies can adopt. It calls for all stakeholders to consider the increasing penetration of mobile networks into rural and remote areas as opening up new opportunities to support the provision of quality health care by broadening knowledge and skills of frontline health care providers, and by supporting obstetric and neonatal emergency care through teleconsultations.

## Abbreviations

CS: Caesarean section
HC: Health centres
ICTs: Information communication technologies
MEM: Method Evaluation Model
WHO: World Health Organization
